# A Comprehensive Review of the Diagnostics for Pediatric Tuberculosis Based on Assay Time, Ease of Operation, and Performance

**DOI:** 10.3390/microorganisms13010178

**Published:** 2025-01-16

**Authors:** Soumya Basu, Subhra Chakraborty

**Affiliations:** Department of International Health, Bloomberg School of Public Health, Johns Hopkins University, Baltimore, MD 21205, USA; sbasu12@jh.edu

**Keywords:** diagnosis, mycobacterium, TB, CRISPR, children, artificial intelligence

## Abstract

Pediatric tuberculosis (TB) is still challenged by several diagnostic bottlenecks, imposing a high TB burden in low- and middle-income countries (LMICs). Diagnostic turnaround time (TAT) and ease of operation to suit resource-limited settings are critical aspects that determine early treatment and influence morbidity and mortality. Based on TAT and ease of operation, this article reviews the evolving landscape of TB diagnostics, from traditional methods like microscopy and culture to cutting-edge molecular techniques and biomarker-based approaches. We examined the benefits of efficient rapid results against potential trade-offs in accuracy and clinical utility. The review highlights emerging molecular methods and artificial intelligence-based detection methods, which offer promising improvements in both speed and sensitivity. The review also addresses the challenges of implementing these technologies in resource-limited settings, where most pediatric TB cases occur. Gaps in the existing diagnostic methods, algorithms, and operational costs were also reviewed. Developing optimal diagnostic strategies that balance speed, performance, cost, and feasibility in diverse healthcare settings can provide valuable insights for clinicians, researchers, and policymakers.

## 1. Introduction

Pediatric tuberculosis (TB) remains a significant global health challenge, with diagnosis often complicated by non-specific symptoms and difficulties in obtaining quality specimens [1]. According to the World Health Organization (WHO), an estimated 1.2 million children and young adolescents (<15 years) fell ill with TB in 2023 with 226,000 deaths globally [2]. Mortality is highest in children under five, with 76% of HIV-negative TB deaths occurring in this age group. Undiagnosed and undertreated cases suffer the highest mortality (96%). HIV co-infection significantly increases mortality risk [3,4]. The COVID-19 pandemic has impacted TB notifications, potentially affecting mortality rates. Underdiagnosis remains a major challenge, with only 49% of estimated pediatric TB cases diagnosed and reported in 2022 [4,5]. WHO has emphasized the need for rapid, accurate diagnostic tools for childhood TB, recognizing that early diagnosis and treatment initiation are crucial for improving outcomes [5].

Diagnosing tuberculosis in pediatric patients is exceptionally challenging due to non-specific symptoms, paucibacillary disease, and difficulties in obtaining adequate sputum samples, especially from young children who cannot expectorate. This underscores the urgent need for novel, non-invasive diagnostic approaches, such as stool-based and blood-based tests [1,5]. Recent years have seen significant advancements in diagnostic technologies aimed at reducing turnaround time (TAT) while maintaining or improving accuracy. For instance, the Xpert MTB/RIF Ultra assay (including stool-based assay), which provides results in about 90 min, has shown improved sensitivity compared to its predecessor in pediatric populations [6,7]. Similarly, newer technologies like the Cepheid MTB-HR, a blood-based test utilizing a three-gene mRNA transcriptomic signature, aim to provide rapid results from easily obtainable samples [1,6]. However, the quest for faster diagnostics raises important questions about the balance between speed, accuracy, and ease of operation in pediatric TB diagnosis. While rapid tests can facilitate quicker treatment initiation, they must be evaluated in the context of their overall clinical utility, including sensitivity, specificity, and applicability in resource-limited settings where the majority of TB cases occur [8,9].

This review provides an up-to-date overview of available diagnostic tools for pediatric TB, exploring their TAT, performance, and application. We discuss traditional methods, such as microscopy and culture, as well as newer molecular techniques and emerging biomarker-based approaches. Additionally, we review how these diagnostic methods perform in different clinical scenarios and patient subgroups, such as HIV-infected children or those with extrapulmonary TB [8]. The data search strategy comprises specific keywords relevant to each section of the manuscript, as well as general keywords like “pediatric tuberculosis”, “TB diagnosis”, “Pediatric TB challenges”, “Cost of TB diagnostic methods”, etc., from the databases PubMed, PubMed Central, Scopus, and Google Scholar. Duplicates were filtered. The search window was primarily set to the timeframe of the last five years and occasionally increased to the last ten years in case of the unavailability of data for specific sections. By critically assessing the premise that ‘faster is better’ in pediatric TB diagnostics, we seek to provide a nuanced understanding of the current landscape and future directions in this crucial area of global health. This review also highlights gaps in current knowledge and areas where further research is needed to improve the diagnosis of TB in children.

## 2. Challenges in Pediatric TB Sampling

The diagnosis of TB in children presents unique challenges due to the age-dependent nature of the disease spectrum and the difficulties in obtaining suitable diagnostic samples. Younger children often exhibit non-specific signs and symptoms of paucibacillary intrathoracic or disseminated infection, making it challenging to differentiate TB from other common childhood illnesses [9]. Symptoms such as fever, cough, and weight loss are not exclusive to TB, complicating the diagnostic process [10]. Moreover, children typically have a lower bacterial load compared to adults, making it harder to detect *Mycobacterium tuberculosis* (Mtb) in sputum samples [1,10].

Various sample collection methods for pediatric TB diagnosis each present their own set of challenges. Expectorated or induced sputum samples, while offering high sensitivity and specificity for Xpert assays, are limited to older children with productive coughs and require specialized equipment and trained personnel [9,11]. Experimental methods like oral swabs and bioaerosols show promise but currently lack optimal diagnostic accuracy or suitable pediatric prototypes [10,11].

The WHO has endorsed stool samples for use with the Xpert MTB/RIF Ultra test, which is particularly beneficial for younger children who struggle to produce sputum [12,13]. The ease of stool collection in children makes it possible to diagnose TB at primary healthcare levels, potentially reducing the need for referrals, although stool processing for sophisticated tests requires some training [12,14]. In some studies, stool-based Xpert testing has shown considerable specificity (98–99%) [14,15]. Stool samples may also be helpful in detecting extrapulmonary TB, which is often missed by smear microscopy [16]. However, stool samples have a range of challenges. The sensitivity of stool-based Xpert testing varies widely (50% to 67%) across studies, with very low sensitivity (2–6%) in clinically diagnosed TB cases compared to culture on respiratory samples [12,14]. Potential for heterogeneity in results of stool-based Xpert testing may occur due to bacterial load, differences in processing methods, or patient populations [17]. These disadvantages limit stool testing as a standalone diagnostic route; rather, it is recommended as an additional specimen rather than a replacement for other diagnostic methods, especially in cases where respiratory samples can be obtained [16,17].

Gastric and nasopharyngeal aspirates are also being increasingly used, showing promising results despite potentially lower sensitivity compared to sputum. However, both sampling types are invasive procedures demanding specific expertise and equipment [18,19]. Blood-based biomarkers in TB diagnosis offer a potential breakthrough, with studies investigating specific cytokines and chemokines [20].

While promising, these alternative methods still face challenges in sensitivity and require further validation in pediatric populations. Nevertheless, they represent important steps towards more child-friendly TB diagnostic approaches. The complexity of sample collection, combined with the non-specific presentation of pediatric TB, underscores the need for innovative diagnostic approaches tailored to the unique needs of the pediatric population [9,10].

## 3. Existing Diagnostic Methods and Gaps

### 3.1. Traditional Diagnostic Methods for Pediatric TB

Microscopy remains the oldest and most widely available diagnostic tool for TB, offering rapid results within minutes to hours [21]. However, its utility in pediatric TB is severely limited due to its low sensitivity, ranging from 10–15% in children [22]. This poor performance is primarily attributed to the paucibacillary nature of pediatric TB, where children often have a low bacterial load in their specimens. A systematic review found that the pooled sensitivity of smear microscopy in children was only 22% (95% CI: 17–28%) compared to culture [13]. Recent advancements, such as LED fluorescence microscopy, have improved sensitivity, with one study reporting an increase from 52% to 73% compared to conventional light microscopy [23]. However, the fundamental limitation persists; low sensitivity makes microscopy an unreliable standalone test for diagnosing TB in children. Empirical TB diagnosis is a common practice in resource-limited settings, based on clinical signs and symptoms without laboratory confirmation. However, the sensitivity of empirical TB diagnosis widely varies from 16% to 44.4% across different studies [24]

Culture-based techniques remain the gold standard for TB diagnosis, offering definitive results and the ability to perform drug susceptibility testing. However, the lengthy turnaround time of 2–8 weeks makes it impractical for rapid diagnosis and timely treatment initiation, which is crucial in pediatric cases. A meta-analysis by Detjen et al. found that the pooled sensitivity of culture in children was 62% (95% CI: 51–73%) compared to a clinical reference standard [25]. Recent innovations like the Microscopic Observation Drug Susceptibility (MODS) assay have reduced the time to 7–10 days, with one study reporting a sensitivity of 87.5% in pediatric samples [26]. However, this is still too long for optimal clinical management. The critical limitation of culture-based methods in pediatric TB lies in the difficulty of obtaining quality specimens in children’s samples.

The Tuberculin Skin Test (TST) has been a cornerstone of TB screening for decades, with a turnaround time of 48–72 h [27]. The tuberculin skin test involves an intradermal injection of purified protein derivative (PPD) to elicit a delayed-type hypersensitivity reaction, which is measured by the size of induration after 48–72 h. However, its utility is hampered by limited specificity, particularly in BCG-vaccinated populations and those exposed to non-tuberculous mycobacteria. A recent meta-analysis reported a pooled sensitivity of 66% (95% CI: 55–76%) and specificity of 75% (95% CI: 65–84%) in children, highlighting its limitations [28]. The TST’s critical shortcoming is its inability to differentiate between active TB and latent infection, a distinction crucial for clinical decision-making in pediatric cases. A study found that the TST had a positive predictive value of only 4% for progression to active TB in children [29].

Interferon-gamma release assays (IGRAs) are blood tests that measure ex vivo T-lymphocyte release of interferon-γ after stimulation by Mtb-specific antigens. IGRAs offer improved specificity over TST, with results available in 24–48 h. A meta-analysis found that IGRAs had a pooled sensitivity of 67% (95% CI: 62–73%) and specificity of 98% (95% CI: 97–99%) in children [28]. However, like TST, IGRAs cannot distinguish between active and latent TB, limiting their diagnostic value. Furthermore, they require specialized laboratory equipment and trained personnel to perform blood collection, processing, and result interpretation, making them less operationally simple for resource-limited settings. Their performance is also suboptimal in young children and immunocompromised patients, who are often at the highest risk for severe TB. A recent study found that IGRA performance was particularly poor in children under 5 years, with indeterminate results in up to 40% of cases [30]. Recent advancements, such as the T-cell activation marker-TB assay, show promise in differentiating active from latent TB, with one study reporting a sensitivity of 83.3% and specificity of 96.8% in children [29]. However, these newer approaches require further validation in diverse pediatric populations.

### 3.2. Molecular Diagnostic Techniques for Pediatric TB

Xpert MTB/RIF is a widely used cartridge-based nucleic acid amplification test (NAAT) for TB diagnosis [31]. It can simultaneously detect TB and rifampicin resistance within approximately 2 h. The assay has a limit of detection of 131 CFU/mL in sputum and detected as few as 10 CFU/mL per sputum sample in 35% of samples with a much longer TAT [32]. The test’s sensitivity for TB detection ranges from 89% to 98% in smear-positive samples and 67% to 75% in smear-negative, culture-positive samples [33]. While Xpert MTB/RIF offers rapid results and requires simple operative expertise, it needs stable electricity and temperature control, which can be challenging in resource-limited settings. It is also limited to detecting resistance to only rifampicin. Additionally, its high equipment cost (~USD 11,000–70,000 based on module type and accessories, excluding maintenance costs) and relatively high cost per test (>USD 20) as compared to smear microscopy limit its widespread use in some contexts [34,35,36]. The Xpert MTB/RIF Ultra assay improves upon its predecessor, the Xpert MTB/RIF, by utilizing two multicopy targets (IS6110 and IS1081) and a larger PCR chamber, resulting in enhanced sensitivity and a lower limit of detection (15.6 CFU/mL) compared to 131 CFU/mL for the original test [37]. The World Health Organization recommends Xpert Ultra as the initial diagnostic test for pulmonary TB in children, as it outperforms traditional methods like smear microscopy and culture, especially in cases with low bacterial loads [38]. However, despite its advantages, Xpert Ultra has faced criticism regarding its specificity, which can decrease as sensitivity increases, leading to potential false positives [37,39].

TrueNAT, a chip-based NAAT for TB detection, provides results in 30–60 min [40]. It involves three main steps, such as sample preparation using a pre-treatment pack, automated DNA extraction using the Trueprep AUTO device (molbio Diagnostics, Goa, India), and PCR amplification/detection using TrueNAT chips and the Truelab Real Time micro-PCR Analyzer [1,5]. Recent studies have shown promising results, with one multicenter evaluation in Cameroon demonstrating a sensitivity of 91% (95% CI: 86–94%) and specificity of 96% (95% CI: 94–97%) compared to culture [33]. However, the performance of TrueNAT can be affected by various factors, including sample quality and operator skill. A study in India found that the rate of invalid MTB results was 5.2% and the rate of indeterminate rifampicin resistance results was 15.3% [34]. While TrueNAT offers quick turnaround times, more data are needed on its performance in diverse settings, particularly for detecting extensively drug-resistant TB.

Line Probe Assay (LPA) can detect TB and resistance to multiple drugs within 1–2 days. It has shown high sensitivity and specificity for detecting rifampicin and isoniazid resistance, especially in smear-positive samples in various studies [41,42]. It uses PCR and reverse hybridization techniques to identify specific DNA sequences on nitrocellulose strips [43]. However, LPA requires sophisticated laboratory infrastructure and skilled technicians. Its inability to be used directly on clinical samples limits its applicability in some settings, particularly at the point of care.

Loop-mediated Isothermal Amplification (LAMP) is a nucleic acid amplification technique that uses 4–6 specially designed primers and a strand-displacing DNA polymerase to amplify target DNA sequences at a constant temperature (60–65 °C). For TB detection, it has a turnaround time of about 1 h [44]. A systematic review and meta-analysis found that LAMP had a pooled sensitivity of 89.6% (95% CI: 85.6–92.6%) and specificity of 94.0% (95% CI: 91.0–96.1%) for pulmonary TB diagnosis [45]. While LAMP offers rapid results owing to its simplicity (to perform and interpret), its lower sensitivity compared to Xpert MTB/RIF to detect drug resistance limit its utility in comprehensive TB management [42,45].

The Lipoarabinomannan (LAM) Antigen Test is a lateral flow immunochromatographic assay that detects mycobacterial LAM in urine samples, providing results within 25–30 min. The test is operationally simple, requiring only a small amount of unprocessed urine and visual interpretation of results, making it suitable for point-of-care use in resource-limited settings [46]. As per the WHO recommendations, LAM testing for TB is considered feasible for HIV-positive patients, especially those with low CD4 counts or severe illness. The newer FujiLAM test shows promise for use in HIV-negative patients but requires optimization with additional steps and higher TAT. A meta-analysis found that the pooled sensitivity of LAM in HIV-positive individuals was 42% (95% CI: 31–55%), increasing to 56% (95% CI: 41–70%) in those with CD4 counts ≤100 cells/μL [47,48].

Next-Generation Sequencing (NGS), for TB diagnosis, particularly targeted NGS (tNGS), is a high-throughput DNA sequencing technology that allows comprehensive detection of drug-resistant Mtb mutants. It involves DNA extraction, library preparation, sequencing, and bioinformatic analysis to identify resistance-conferring mutations across multiple genes simultaneously. Recent studies have demonstrated that culture-free tNGS can provide accurate sequencing results directly from clinical samples, offering high diagnostic accuracy for first-line drugs, injectable drugs, and fluoroquinolones while also showing promise for newer drugs like bedaquiline and delamanid. With a TAT of 2–3 days, NGS offers higher comprehension in terms of resistance profiling compared to phenotypic drug susceptibility testing, however, its implementation in routine TB diagnostics faces challenges such as the need for specialized equipment, complex sample preparation, sophisticated bioinformatics analysis, and higher costs per sample (USD 75–200 for NGS). However, efforts are ongoing to simplify workflows and reduce turnaround times, making it increasingly feasible for implementation in reference laboratories in high-burden settings [49,50,51].

The TAM-TB (T-cell Activation Marker-TB) assay is a novel immunodiagnostic test for TB that measures the expression of activation markers (CD38, HLA-DR, Ki67) and a maturation marker (CD27) on Mtb-specific CD4 T-cells using flow cytometry [52]. Requiring only minimal fresh blood, it shows promise for diagnosing extrapulmonary TB and in pediatric cases, with results available within 24 h [53]. The assay can distinguish between active and latent TB infection and potentially monitor treatment response [52,53]. However, it requires specialized equipment and trained personnel, limiting its current use as a point-of-care test. While promising, further validation in larger cohorts and diverse populations is needed before widespread clinical implementation [52].

**Figure 1:**
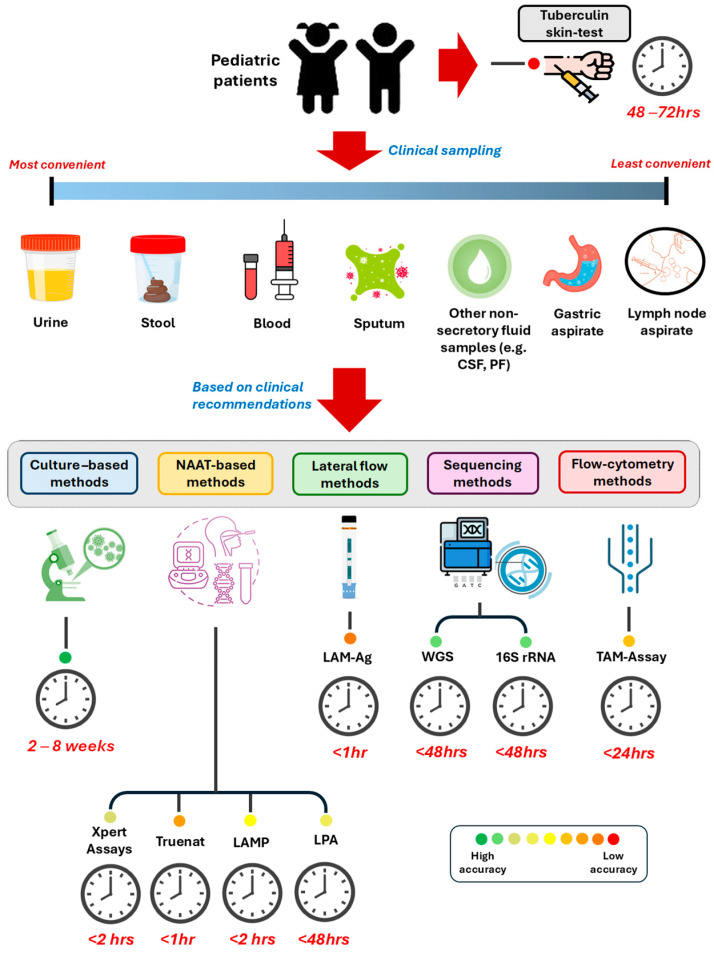
Overview of recommended pediatric TB diagnostic samples and tests based on the types, turnaround time, and accuracy.

The combination of centralized laboratory-based testing and decentralized point-of-care testing is increasingly recognized as an effective approach to TB diagnosis [54]. This strategy can help balance the need for rapid results with comprehensive drug resistance profiling. For example, using Xpert MTB/RIF or TrueNAT at the point of care for initial diagnosis, followed by more comprehensive testing like LPA or NGS at reference laboratories for drug resistance profiling, could improve overall TB care cascades [55]. An overview of the recommended diagnostic methods is given in Figure 1 based on speed, ease of operation, and accuracy. The ongoing development and refinement of these diagnostic tools continue to play a crucial role in the global fight against TB (Table 1). 

### 3.3. Emerging Approaches in Pediatric TB Diagnosis

The landscape of TB (TB) diagnostics is rapidly evolving, with several innovative approaches showing promise for improved detection, particularly in challenging populations such as children [60]. An overview of recent developments and proof-of-concept studies is summarized.

The Cepheid MTB-HR (Host Response) assay represents a significant advancement in TB diagnosis, utilizing a blood-based host response signature. With a turnaround time of approximately 90 min and the ability to use easily obtainable fingerstick blood samples, it shows promise for pediatric TB diagnosis [61]. A recent multicenter study conducted across South Africa, Uganda, and Vietnam demonstrated encouraging results in children, achieving a sensitivity of 83% and specificity of 84% for TB detection [62]. However, further validation in diverse pediatric populations is needed to establish its place in diagnostic algorithms, especially in high-burden, low-resource settings.

CRISPR-based detection methods are at the forefront of innovative TB diagnostics. These techniques offer results within 1–2 h and show promise for ultrasensitive detection of TB DNA [63]. A recent study by Zhang et al. developed a CRISPR/Cas12a-based assay combined with recombinase-aided amplification (RAA) that could detect Mtb with a limit of detection as low as 3.13 CFU/mL [64]. This method, termed TB-CRISPR, demonstrated a sensitivity of 88.3% and specificity of 94.0% when compared to BACTEC 960 culture, with a total detection time of less than 1.5 h [63,64,65]. A CRISPR/Cas13a assay showed a low limit of detection of one target sequence copy/μL and provided superior sensitivity (97.4%) compared to acid-fast bacilli (48.5%) and mycobacterial culture (71.6%) assays in clinical samples. The turnaround time for this assay was not explicitly stated but is likely within the 1–2-h range typical of CRISPR-based methods [65,66]. A novel application of CRISPR technology involves the detection of circulating cell-free TB DNA (Mtb-cfDNA) in serum. A study demonstrated high sensitivity (100%) and specificity (94%) in detecting microbiologically and clinically confirmed TB cases in a predominantly HIV-negative adult cohort. Importantly, this method also showed promise in pediatric populations, with 83% sensitivity and 95% specificity in a cohort of children, including all cases of extrapulmonary TB [66].

A report by MacLean et al. identified a three-protein biosignature (C-reactive protein, transthyretin, and complement factor H) that could differentiate TB from other diseases with 86% sensitivity and 84% specificity [20]. This approach, which uses a blood test, has a potential turnaround time of a few hours and could be particularly valuable for children who often struggle to produce sputum samples. Metabolomic approaches are also showing promise. A recent study by Manyelo et al. identified a 7-marker serum metabolomic biosignature that showed 80% sensitivity and 89% specificity for TB diagnosis in children [67]. While these biomarker-based approaches are still in the discovery or early validation phases, they offer the potential for rapid, non-sputum-based diagnostics with turnaround times potentially as short as a few hours once fully developed [68]. While these innovative approaches show great promise, particularly for challenging diagnostic scenarios like pediatric and extrapulmonary TB, they are still in various stages of development, validation, or at the proof-of-concept stage. Further large-scale studies in diverse populations are needed to establish their accessibility, clinical utility, and cost-effectiveness before they can be widely implemented in TB diagnostic procedures [69].

Portable chest X-ray (CXR) systems combined with computer-aided detection (CAD) software can improve access to screening in remote areas. CXR has higher sensitivity and specificity than symptom screening alone and can potentially reduce the number and costs of follow-on diagnostic tests [70,71]. Recent advances in machine learning and AI-based methods have significantly improved TB diagnosis, particularly through the analysis of medical imaging and molecular data [72]. Some of the popular tools are provided in Table 2. Deep learning models, especially convolutional neural networks (CNNs) such as ResNet, VGG, and AlexNet, have demonstrated high accuracy in detecting TB from chest X-rays, with some studies reporting area under the receiver operating characteristic curve (Area Under Curve) values of 0.99 or higher [73]. For instance, a ResNet-based AI system achieved 96.73% accuracy in TB detection from chest X-rays, outperforming other models. These AI systems have shown comparable or even superior performance to radiologists in TB detection tasks [74,75]. Additionally, AI-based radiographic extent analysis, such as the one developed using OpenCV for visualization, has emerged as a significant predictor of treatment success and culture conversion in pulmonary TB. Beyond image analysis, machine learning approaches like support vector machines (SVMs) and random forests have been applied to genetic data for rapid detection of drug-resistant TB strains. Tools like Xpert MTB/RIF assay, combined with AI analysis of its cycle threshold (Ct) values, offer promising avenues for improving TB diagnosis and treatment monitoring [75,76]. AI-CAD is seen as a potential solution to find undetected tuberculosis cases by reducing the need for costly confirmatory diagnostics. However, beyond the accuracy, the implementation of CAD in TB diagnosis globally requires manufacturer-independent validation, cost-effective resource allocation, uptake by state authorities for integration into existing TB diagnostic systems, and ensuring equitable access. In resource-constrained settings, CAD can address the scarcity of skilled radiologists, offer a cost-effective screening alternative, and support active case finding in HIV-endemic regions. High-income countries benefit from CAD’s ability to augment diagnostic accuracy and streamline radiologist workflows, thereby enhancing the rate of diagnosis. However, implementation is subject to methodological ease, the need for context-specific threshold determination, substantial investment, and training [77].

### 3.4. Balancing Speed, Accuracy, and Cost of Diagnosis

Recent studies have highlighted the critical role of cheap and rapid diagnosis in improving patient outcomes and reducing disease transmission. Shorter TATs could lead to earlier treatment initiation, potentially reducing morbidity and mortality associated with TB in children [9]. Diagnostic cost is one of the most critical factors that limit the implementation of rapid and sophisticated tests. A recent analysis of moderate complexity automated nucleic acid amplification tests (MC-NAATs) for TB diagnosis revealed that the base-case per-test cost was USD 18.52 (range: USD 13.79–40.70) for lower throughput (LT) tests and USD 15.37 (range: USD 9.61–37.40) for higher throughput (HT) tests [81]. These costs were most sensitive to the number of testing days per week, equipment costs, and TB-specific workloads. Generally, HT NAATs were more cost-effective at all testing volume levels, but LT tests could be cheaper at lower testing volumes (fewer than 2000 per year) if the durability of the testing system was markedly better or equipment costs were lower [81].

In high multidrug-resistant TB (MDR-TB) settings, such as Moldova, the average diagnostic cost per patient suspected of TB, including diagnosis of TB and TB drug resistance, was estimated at USD 82 [82]. This study provided empirical estimates for various diagnostic tests, including sputum smear microscopy (SSM), Löwenstein–Jensen (LJ) solid culture, BACTEC MGIT, Xpert MTB/RIF, and line-probe assays (LPAs). The per-test costs ranged from USD 8.15 for SSM to USD 30.75 for Xpert MTB/RIF [82].

A cost and affordability analysis of TB-LAMP and Xpert MTB/RIF assays in Vietnam and Malawi demonstrated that the weighted average per-test cost of nationwide implementation was between USD 14.37–15.85 for TB-LAMP and USD 20.06–26.86 for Xpert [83]. This study highlighted that both NAATs would account for a significant portion of or exceed the national TB program budget if complete nationwide roll-out to peripheral laboratories were considered.

The balance between speed and accuracy in different settings remains another crucial consideration. The trade-offs between rapid results and diagnostic accuracy continue to be a subject of intense debate. While faster results can lead to quicker treatment initiation, the potential for false-positive or false-negative results must be carefully considered. A report explored the clinical implications of false-positive results from rapid molecular tests, highlighting the risks of unnecessary treatment and the psychological impact on patients and families [84]. Conversely, false-negative results can lead to delayed diagnosis and treatment, potentially resulting in disease progression and continued transmission. A recent meta-analysis demonstrated that the optimal diagnostic strategy varies depending on the local TB prevalence, available resources, and healthcare infrastructure [32]. In high-burden, resource-limited settings, rapid point-of-care tests with moderate sensitivity may be preferred over highly accurate but lengthy laboratory-based methods. Conversely, in low-burden settings with more resources, a combination of rapid screening tests followed by confirmatory molecular assays might be more appropriate [85]. Therefore, future research should focus on developing innovative, cost-effective diagnostic strategies that balance speed, accuracy, ease of use, and accessibility to improve TB detection and patient outcomes across various resource settings.

## 4. Future Directions

Sample collection and implementation of state-of-the-art diagnostic tools in resource limited settings are the major challenges owing to infrastructure, cost, and specialized skills required [13,85]. Active case finding (ACF) has shown potential for increasing early detection of TB cases in underserved communities. Effective RDTs for TB detection, along with capacity-building for healthcare workers, would facilitate ACF implementation in LMICs [86]. In addition, creating centralized “hubs” that perform advanced diagnostics like whole genome sequencing for each country or region can help overcome infrastructure and cost barriers. This approach requires developing efficient sample transport systems [87]. For economic accessibility of the diagnostic tools, negotiating with manufacturers and exploring bulk purchasing options can help reduce costs. Building bioinformatics and laboratory capacity, besides investing in training programs, can develop local expertise to expedite the process [13,85].

Home-based TB diagnosis is emerging as a promising approach to improve case detection and reduce barriers to TB evaluation. A proof-of-concept study in Eastern Cape, South Africa, found in-home testing using GeneXpert Edge highly acceptable, with a 98% consent rate and 96% result delivery. It significantly increased testing rates (47% vs. 13%) and reduced result turnaround time (0 vs. 16.5 days median) compared to clinic-based testing [88]. However, a Uganda study using SMS-facilitated evaluation showed implementation challenges [89]. Recent high-impact literature on pediatric TB diagnosis and management has led to several key recommendations for clinicians, researchers, and policymakers to improve pediatric TB conditions worldwide [1,90]. Policymakers play a vital role in improving pediatric TB conditions. Updating clinical guidelines to incorporate new evidence-based diagnostic algorithms and novel diagnostic tools is essential. Investing in health system strengthening, including improved infrastructure, supply chains, and training programs, is necessary to support the implementation of new diagnostic tools. Promoting integrated care approaches by encouraging the integration of TB services with other child health programs can improve case detection and treatment initiation. Supporting research and development through resource allocation for pediatric-specific TB diagnostics and treatments is crucial. Finally, addressing social determinants by implementing policies to tackle poverty, malnutrition, and other social factors that contribute to TB vulnerability in children is of utmost importance.

To summarize, combating pediatric TB demands a multifaceted strategy: simple and rapid tests, non-invasive sampling, and integration with child health services. Feasible and effective innovative diagnostic tools, including detection of drug resistance and expanded contact tracing, can enhance detection and prompt treatment. Success hinges on political engagement, funding advocacy, and global collaboration—a holistic approach to revolutionizing pediatric TB care and saving young lives worldwide.

## 5. Conclusions

Implementing fast, simple, and accurate TB diagnostic tools in resource-limited settings of LMICs requires a multifaceted approach that combines technological innovations, capacity building, and health system strengthening. By adopting these strategies and continually evaluating their effectiveness, endemic regions, especially LMICs, can work towards improving TB diagnosis and moving closer to achieving global TB elimination goals.

## Figures and Tables

**Table 1:** Existing diagnostic methods for TB summarizing the technology, targets, accuracy, and recommendations [56,57,58,59].

Test	Technical Simplicity	Technology	Time Taken	Age	Symptoms and Medical History	Target Population			Sample	**Accuracy**	**Recommendations**
						Pediatric	Adolescents	Adults			
^±^ Xpert MTB/RIF and/or Xpert Ultra	Moderately simple; requires moderate training	qPCR	<2 h	>15 years	Signs and symptoms of pulmonary TB	-	√	√	Sputum	High	Initial diagnosis strongly recommended
				<15 years	Signs and symptoms of pulmonary TB	√			Sputum, gastric aspirate, nasopharyngeal aspirate, and stool	Moderate to Low	Strongly recommended
				>15 years	Signs and symptoms of pulmonary TB and without a prior history of TB (≤5 years) or with a remote history of TB treatment (>5 years since end of treatment)	-	√	√	Sputum	High	Initial diagnosis strongly recommended
				>15 years	Signs and symptoms of pulmonary TB and with a prior history of TB and an end of treatment <5 years	-	√	√	Sputum	High	Initial diagnosis strongly recommended
				All	Signs and symptoms of TB meningitis	√	√	√	Cerebrospinal fluid (CSF)	Moderate to Low	Strongly recommended
				All	Signs and symptoms of extrapulmonary TB	√	√	√	Lymph node aspirate, lymph node biopsy, pleural fluid, peritoneal fluid, pericardial fluid, synovial fluid, or urine specimens	Moderate to Low (Strong for rifampicin resistance)	Conditionally recommended (strongly recommended for Xpert MTB/RIF)
				All	Signs and symptoms of disseminated TB (HIV-positive)	√	√	√	Blood	Moderate to Low	Conditionally recommended
				>15 years	General population who had either signs or symptoms of TB or chest radiograph with lung abnormalities or both		√	√	Blood	Moderate to Low	Conditionally recommended
^±^ TrueNAT MTB, MTB plus, (under development: MTB-Ultima, MTB-INH, MTB-BDQ, MTB TB-COVID-19)	Moderately simple; requires moderate training	Micro RT-PCR	<1 h	All	With signs and symptoms of pulmonary TB	√	√	√	Sputum	Moderate	Conditionally recommended
^±^ TrueNAT MTB-RIF Dx	Moderately simple; requires moderate training			All	With signs and symptoms of pulmonary TB and a TrueNAT MTB or MTB Plus positive result	√	√	√	Sputum	Low	Conditionally recommended
^±^ Moderate complexity automated nucleic acid amplification tests (NAATs)	Requires highly trained facility/manpower	High-throughput molecular PCR	6–8 h	All	Signs and symptoms of pulmonary TB	√	√	√	Respiratory samples	Moderate	Conditionally recommended (also for isoniazid and rifampicin resistance)
^±^ Loopamp MTBC assay	Simple with moderate training	Loop-mediated isothermal amplification	<2 h	>15 years	Signs and symptoms consistent with TB	√	√	√	Sputum	Low	Conditionally recommended
				>15 years	Necessary further testing of sputum smear-negative specimens	√	√	√	Sputum	Low	Conditionally recommended
^±^ LAM Ag assay	Simple with minimal instructions	Lateral flow urine lipo-arabino-mannan assay	<1 h	All	In inpatient settings → HIV-positive adults and children with signs and symptoms of TB, CD4 cell count of less than 200 cells/mm^3^	√	√	√	Urine	Moderate to Low	Conditionally recommended
				All	In outpatient settings → HIV-positive adults and children with signs and symptoms of TB, CD4 cell count of less than 100 cells/mm^3^	√	√	√	Urine	Low	Conditionally recommended
^±^ First-line line-probe assay (LPAs)	Requires highly trained facility/manpower	Multiplex PCR+ DNA strip reverse hybridization assay	<48 h	All	Sputum smear-positive specimen or a cultured isolate of Mtb complex (MTBC)	√	√	√	Sputum	Moderate	Conditionally recommended (rifampicin/isoniazid resistance)
Second-line line-probe assays (SL-LPAs) *	Requires highly trained facility/manpower	Multiplex PCR+ DNA strip reverse hybridization assay	<48 h	All	Confirmed MDR/RR-TB	√	√	√	Sputum	Moderate to low	Conditionally recommended (Fluoroquinolone resistance detection)
^±^ High complexity reverse hybridization-based NAATs	Requires highly trained facility/manpower	Multiplex PCR+ DNA strip reverse hybridization assay (targeting the entire *pncA* gene)	Variable (<24 h)	All	Bacteriologically confirmed TB	√	√	√	TB culture isolates	Low	Conditionally recommended (specialized for pyrazinamide resistance)
Next-generation sequencing	Requires highly trained facility/manpower	Whole genome/targeted sequencing	<48 h	All	NA	√	√	√	Sputum, TB culture isolates	High	NA
TAM TB assay *	Requires highly trained facility/manpower	Flow cytometry/TB specific biomarkers CD38/CD27	<24 h	All	NA	√	√	√	Blood	Moderate to High	NA

* Not available commercially; ^±^ WHO recommended; ‘√’ Applies to this section; ‘-’ Does not apply to this section.

**Table 2:** Some machine learning/AI-based tools designed to escalate TB diagnosis.

Tool	Accuracy	Input	Key Feature	References
CAD4TB (version 7)	94% sensitivity and 84% specificity	Chest X-rays	Includes modules for registration, symptom screening, X-ray imaging, and integration with GeneXpert systems	[78]
EfficientNetB3 (https://huggingface.co/google/efficientnet-b3)	High performance (highest Area Under Curve of 0.999)	Chest X-rays	A convolutional neural network structure that can accurately detect mislabeled and missed findings	[73]
qSpot-TB (https://www.qure.ai/global-health)	96% sensitivity	Chest X-ray analysis	Received FDA breakthrough device designation	[74]
InferRead DR (version 2)	90% sensitivity and 70.4% specificity	Chest X-ray analysis	Screening time is <1 min, no subsequent validation suggested	[75]
Lunit INSIGHT (https://www.lunit.io/en/products/mmg)	~89% sensitivity	Chest X-ray analysis	Clinical evaluations worldwide show promise in conspicuity among other tools	[75]
JF CXR-1 (http://intl.jfhealthcare.com/en/product.html)	94% sensitivity	Chest X-rays	Clinical evaluations worldwide show promise working under limited resources	[75]
qXR (https://www.qure.ai/product/qxr)	~91% sensitivity	Chest X-rays	Received FDA/CE clearances	[79]
Google Health AI system (https://health.google/health-research/imaging-and-diagnostics/)	Yet to be determined	Chest X-rays	A deep learning-based system capable of personalized health management	[80]

## Data Availability

No new data were created or analyzed in this study.
